# An ecological approach to caregiver burnout: interplay of self-stigma, family resilience, and caregiver needs among mothers of children with special needs

**DOI:** 10.3389/fpsyg.2025.1518136

**Published:** 2025-02-19

**Authors:** Catherine So-Kum Tang, Isaac Chun-Yeung Yu, Kai-hang Ng, Helen Sin-Hang Kwok

**Affiliations:** ^1^Department of Counselling and Psychology, Hong Kong Shue Yan University, North Point, Hong Kong SAR, China; ^2^Mrs Dorothy Koo & Dr Ti Hua Koo Centre for Interdisciplinary Evidence-Based Practice and Research, Hong Kong Shue Yan University, North Point, Hong Kong SAR, China

**Keywords:** parental caregiver burnout, mothers with SN children, self-stigma, family resilience, caregiver needs support

## Abstract

**Introduction:**

Using an ecological framework, this study investigated how individual perceptions (self-stigma), family dynamics (family resilience), and external support systems (caregiver needs) interacted with each other to impact caregiver burnout among mothers of children with special needs (SN) in Hong Kong.

**Methods:**

A total of 250 Chinese mothers of children with SN in Hong Kong completed an online survey.

**Results:**

Pearson correlation analyses indicated that high levels of caregiver burnout were significantly related to high levels of self-stigma, high levels of unmet caregiver needs, and low levels of family resilience. Multiple regression analysis revealed significant main effects and a 3-way interaction effect of these three factors on caregiver burnout. The significant 3-way (self-stigma X family resilience X caregiver needs) interaction effect showed that in conditions of low self-stigma, the highest level of caregiver burnout was found among mothers with low family resilience and high caregiver needs. Conversely, in conditions of high self-stigma, the highest level of burnout was observed among mothers with high family resilience and high caregiver needs.

**Discussion:**

The findings of this study underscore the necessity of a holistic and culturally sensitive approach to effectively reducing the caregiving burden among Chinese mothers of children with SN. Addressing caregiver burnout in these mothers requires simultaneous efforts to reduce self-stigma through psychological support, educate families to manage expectations and alleviate pressure, and enhance society resources to meet caregiver needs.

## 1 Introduction

Parental caregiving burnout is a state of physical, emotional, and mental exhaustion that occurs when parents are overwhelmed by the demands of caring for their children. This condition is characterized by chronic fatigue, feelings of helplessness, and a sense of being emotionally drained (Kwiatkowski and Sekulowicz, 2017; Hubert and Aujoulat, 2018). Burnout can significantly reduce parents’ physical and mental well-being (Maslach and Leiter, 2016), leading to tiredness, stress, withdrawal, anxiety, depression, and even suicidal thoughts (Lheureux et al., 2016; Koutsimani et al., 2019). These symptoms impact their ability to provide effective care for their children and other family members (Alves et al., 2019; Gérain and Zech, 2019). About 5–9% of parents experience caregiving burnout, with higher rates among parents of children with special needs (SN).

Research shows that parental caregiving burnout is influenced by individual, interpersonal, community, and policy/cultural factors (see review by Ren et al., 2024). This study aims to explore the multifaceted factors contributing to parental caregiving burnout in Hong Kong using an ecological framework (Bronfenbrenner, 1979; Bronfenbrenner and Evans, 2000). This framework posits that individuals’ development and experience are influenced by a series of interconnected systems, ranging from personal attributes and immediate surroundings (e.g., family) to broader society structures (Cheng and Lai, 2023; Abeasi et al., 2024; Lobo et al., 2024). It provides a comprehensive understanding of caregiver burden, as it considers multiple levels of influence on caregivers’ experience. Specifically, this study selected individual perceptions (self-stigma), family dynamics (family resilience), and external support systems to fulfil caregiver needs (caregiver needs) as the major study variables and examined how these factors interact to contribute to or mitigate parental caregiving burnout. As mothers often bear the primary caregiving responsibilities in Chinese societies (Huang et al., 2021; Sim et al., 2023), this study will focus on examining caregiving burnout among mothers of children with SN in Hong Kong. For the present study, children with SN refers to children who require additional supporting during education and development due to their physical, developmental, or emotional challenges such as autism, hyperactivity disorder, and chronic illnesses or disabilities (Thurston et al., 2011; Roskam et al., 2021; Findling et al., 2022; Roskam and Mikolajczak, 2023).

At the individual level of the ecological framework (Bronfenbrenner, 1979; Bronfenbrenner and Evans, 2000), parents’ self-stigma could be classified as an internal negative societal stereotype that creates distress through the interaction within individual’s and other external factors. Self-stigma significantly contributes to caregiving burnout among mothers of children with SN by intensifying mothers’ feelings of inadequacy, shame, and guilt. When mothers internalize societal stereotypes and prejudices about having children with SN, they may develop negative beliefs about themselves, leading to chronic emotional stress and exhaustion (De Leersnyder et al., 2013; Eaton et al., 2019; Awan et al., 2022). This emotional burden is compounded by cultural expectations, particularly in Chinese societies, where there is a strong emphasis on maintaining social harmony and suppressing personal distress. Mothers may feel pressured to hide their struggles and not seek help, fearing judgment or social repercussions, exacerbating their stress and isolation. Traditional gender roles further intensify this emotional burden. In many Chinese societies, mothers are often expected to prioritize caregiving responsibilities over personal and professional aspirations. In Hong Kong, for instance, mothers frequently juggle dual caregiving and work responsibilities while adhering to cultural expectations of self-sacrifice and emotional restraint (Sun, 2013). Despite a gradual shift toward more egalitarian gender attitudes, studies show that Chinese mothers still bear the major responsibilities of caregiving, especially when their children exhibit behavioral or emotional challenges (Shek et al., 2019; MacMullin et al., 2021; Zhang, 2022). Mothers experiencing self-stigma may neglect their own needs (Chan et al., 2018), further depleting their physical and emotional resources. This neglect can accelerate the onset of caregiving burnout, as they struggle to maintain their well-being while caring for their children. Additionally, self-stigma can lead to a perceived lack of social support, even if support is available. This perception can reduce help-seeking behavior and increase feelings of isolation (Lannin and Bible, 2022; Prizeman et al., 2023).

At the interpersonal level of the ecological framework (Bronfenbrenner, 1979; Bronfenbrenner and Evans, 2000), family resilience, i.e., the collective strength and adaptability of a family unit in the face of stress and adversity is crucial for managing stress and preventing caregiving burnout among parents of children with SN. Resilient families, develop problem-solving skills and adaptive routines, seek external resources like community support and professional counseling, and share caregiving responsibilities to prevent burnout (Tang et al., 2024). Positive family dynamics, characterized by strong bonds and mutual respect, create a supportive environment (Thomas et al., 2017). In contrast, caregivers in low resilient families often social isolation and experience heightened stress (Cheng and Lai, 2023; Cici and Özdemir, 2024; Patty et al., 2024). Furthermore, family resilience also fosters a sense of purpose and meaning in caregiving roles, increasing motivation and reducing burnout. This is reinforced through family rituals and recognition of caregivers’ efforts. Additionally, resilient families are flexible and adaptable, managing the unpredictable nature of caring for children with SN. In Chinese societies such as Hong Kong, family resilience is rooted in values such as respect for elders and filial piety, which promote family unity and solidarity (Yu and Fang, 2022). These cultural values help families adapt and thrive despite adversity by fostering strong bonds and mutual support (Cheng and Lai, 2023). Chinese families often exhibit higher levels of resilience due to collective values and a strong sense of duty towards family members(Chen and Yu, 2023; Gao et al., 2023). This emphasis on family cohesion and collective well-being in Chinese culture plays a significant role in enhancing family resilience and helping families manage stress and challenges effectively.

At the community level of the ecological framework (Bronfenbrenner, 1979; Bronfenbrenner and Evans, 2000), a lack of social and community support will exacerbate the challenges faced by family caregivers. In regions like Hong Kong and other high-income countries in Asia, inadequate diagnostic assessment and intervention services for children with developmental disabilities further complicate the situation (Tait et al., 2016; Sandbank et al., 2023; Smythe et al., 2024). Caregivers in these contexts often face significant barriers to accessing essential health and community resources, leading to increased stress and burnout. Addressing caregiver needs is critical in alleviating stress, especially for those caring for children with SN. Tan (2015) categorizes these needs into direct care, access to useful information, financial assistance, and social, emotional, and community support. When communities fail to provide adequate resources or accessible services, caregivers struggle with the daily demands of caregiving, including lack of respite care, insufficient information, and limited social and emotional support. This exacerbates stress levels, as navigating complex healthcare and education systems without professional guidance can be overwhelming. Research consistently shows that insufficient social support significantly predicts increased caregiver stress (Neely-Barnes and Dia, 2008; Casagrande and Ingersoll, 2021; Rakap and Vural-Batik, 2023). Cheng and Lai (2023) emphasize that without proper external support, caregivers may adopt ineffective coping strategies, worsening their stress and negatively affecting their child’s development. These findings highlight the importance of robust social connections and community support systems in mitigating caregiver burnout.

### 1.1 Purposes of the present study

There is a growing recognition of the need for an ecological approach to understand caregiver burnout (Ren et al., 2024), particularly challenges faced by family caregivers of children with SN (Abeasi et al., 2024; Lobo et al., 2024). Existing research often overlooks cultural variations, especially in Asian communities, where cultural expectations and societal norms, like self-stigma and family dynamics, significantly shape caregiver stress and coping mechanisms. This study focuses on mother caregivers within the Chinese context in Hong Kong. It adopts an ecological framework (Bronfenbrenner, 1979; Bronfenbrenner and Evans, 2000) to examine diverse influences on caregiver stress and burnout, considering stressors, personality traits, family dynamics, and broader social environment. Specifically, this study investigates how individual perceptions (self-stigma), family dynamics (family resilience), and external systems (external support to caregiving needs) interact to contribute to or mitigate caregiving burnout among mothers in Hong Kong.

Hypothesis 1: High levels of self-stigma are related to caregiver burnout among mothers of children with SN.

Hypothesis 2: Low levels of family resilience are related to caregiver burnout among mother caregivers of children with SN.

Hypothesis 3: High levels of unmet caregiver needs are related to caregiver burnout among mother caregivers of children with SN.

Hypothesis 4: Self-stigma, family resilience, and unmet caregiver needs interact with each other to influence caregiver burnout among mothers of children with SN.

## 2 Method

### 2.1 Participants

This study recruited 250 Chinese mothers of children with SN in Hong Kong. The inclusion criteria required participants to be a parent and primary caregiver of at least one child with SN aged between 3 and 12 years old. This age range covers the school age from kindergarten to primary school. Children at this developmental period show a high caregiving demand from their caregiver (Rotberg et al., 2020), aligning with the study’s objective to assess the parental caregiver burnout during these formative years. For this study, SN encompass conditions such as intellectual disabilities, physical disabilities, visual impairments, hearing impairments, attention-deficit/hyperactivity disorder (ADHD), autism spectrum disorder (ASD), speech and language impairments, and various learning difficulties.

Participants ranged in age from 28 to 52 years, with an average age of 39.6 years. Approximately half had completed secondary education (48.4%), and nearly half had attained tertiary education (48%). Most participants were married (87.2%), and 25.2% were caring for more than one child with SN. The average age of the target child with SN was 7.41 years, and about 75.2% of these children were male. On average, the target children had three SN conditions. The most frequently reported SN conditions among the target children were attention-deficit hyperactivity (83.2%), speech and language impairment (65%), other special learning difficulties (61.2%), and autism spectrum disorder (60%). Table 1 provides detailed socio-demographic information for the participants and their target children with SN.

**Table 1:** Demographic information of participants (*N* = 250).

	n	%	M (SD)
Age (in years)			39.60(4.83)
Monthly household income (HKD)			
<$10,000	17	6.8	
$10,000 to $39,999	149	59.6	
$40,000 to $79,999	40	16.0	
≥$80,000	18	7.2	
Missing	26	10.4	
Employment status			
Full-time	47	18.8	
Part-time	40	16.0	
Unemployed/retired	163	65.2	
Relationship status			
Single	4	1.6	
Married	218	87.2	
Separated/divorced/widowed	28	11.2	
Education level			
Primary education	8	3.2	
Secondary education	121	48.4	
Tertiary education	121	48.4	
Number of special needs children bearing			
1	187	74.8	
2	57	22.8	
3	6	2.4	
Age of the targeted special needs child (in years)			7.41 (2.04)
Sex of the targeted special needs child			
Male	188	75.2	
Female	62	24.8	

### 2.2 Measures

Caregiver burnout was measured using the Emotional Fatigue Measure (EFM) by Ilies et al. (2015). The EFM items were derived from the emotional exhaustion scale of the Maslach Burnout Inventory (Maslach and Jackson, 1986), which has a validated Chinese version (Tang, 1998). The EFM items were worded with special references to caregiving of the target children. The EFM consists of a 4-item, 5-point Likert scale, with responses ranging from 1 (strongly disagree) to 5 (strongly agree). Higher scores on the EFM indicate greater caregiver burnout. Ilies et al. (2015) found that the scale had high internal consistency. In the current study, the Cronbach’s Alpha for the EFM is 0.78.

Self-stigma was measured using the Parents’ Self-Stigma Scale (PSSS) by Eaton et al. (2019). Developed by Eaton et al. (2019), this scale assesses parents’ feelings of self-blame, self-shame, and self-belief. It includes 11 items on a 5-point Likert scale, with responses ranging from 1 (never) to 5 (almost all the time). Higher scores on the PSSS indicate a high level of self-stigma among parents. Eaton and his colleagues reported high internal consistency for this scale. In the present study, the Cronbach’s Alpha for the PSSS is 0.86.

Family Resilience was measured by the Family Resilience Scale (FRS-16: Chow et al., 2022), which is a shortened Chinese version of the Family Resilience Assessment Scale (Sixbey, 2005). The FRS-16 includes items on family communication and connectedness, positive framing, and seeking external support. The scale consists of 16 items rated on a 4-point Likert scale, with values ranging from 1 (strongly disagree) to 4 (strongly agree). Higher scores on the FRS-16 denote a higher level of family resilience. Chow et al. (2022) found that this scale displayed high internal consistency. The current study’s Cronbach’s Alpha for the FRS is 0.85.

Caregiver Needs was measured by the Chinese version of the Caregiver Needs Scale (CNS) in assessing the needs of caregivers for children with disabilities (Tan, 2015). The scale measures various areas of need, including children’s daily care, useful information, social, emotional and community support, medical and professional services, and financial needs. It consists of 20 items on a 5-point Likert scale, ranging from 1 (not at all needed) to 5 (extremely needed). Higher scores on the CNS indicate a high level of unmet caregiver needs and a greater demand for both tangible professional support and intangible social–emotional support for caregivers. In the current study, the Cronbach’s Alpha for the CNS is 0.94.

### 2.3 Procedures

Participants were recruited through online and printed advertisements, posters, and invitation letters sent to local organizations, schools for children with SN, and related support groups. These materials included information on the study’s purpose, a brief description of the research design, and the inclusion and exclusion criteria of participants. Participants completed an online questionnaire. A consent form was displayed on the first page of the online survey, informing participants of their rights and the study’s objectives. Participants were also informed that they could withdraw from the study at any time without penalty. Confidentiality was assured, with personal identities not identifiable, and data used solely for research purposes. Participants gave their consent by clicking the ‘Continue to next page’ button on the first page of the online survey. They received a 50 HKD (approximately 6 USD) supermarket e-voucher for their participation. The completed surveys were then exported from the platform, and the data was stored on a local drive accessible only to the research team. The research was approved by the Human Research Ethics Committee of the authors’ affiliated university.

## 3 Results

Pearson correlation analyses were conducted to determine the relationship between two variables, and their results are summarized in Table 2. The findings supported Hypotheses 1 to 3, showing that high levels of caregiver burnout were significantly related to high levels of caregiver self-stigma (*r* = 0.46, *p* < 0.01), high levels of external caregiver needs (*r* = 0.29, *p* < 0.01), and low levels of family resilience (*r* = −0.36, *p* < 0.01). Additionally, a greater number of target children’s disabilities was significantly related to higher levels of caregiver burnout (*r* = 0.18, *p* < 0.01), mothers’ self-stigma (r = 0.21, *p* < 0.01), and external caregiver needs (*r* = 0.23, *p* < 0.01). However, the ages of parents and the target children were not significantly related to any of these variables (*p* > 0.05).

**Table 2:** Descriptive statistics correlation analyses (*N* = 250).

	M (SD)	Range	1	2	3	4	5	6	7
(1) Age	39.60 (4.83)	28–52	-						
(2) Age of child	7.41 (2.04)	3–12	0.38**	-					
(3) Burnout	13.92 (3.21)	5–20	0.02	−0.03	-				
(4) Self-stigma	29.00 (7.27)	12–55	0.04	−0.01	0.46**	-			
(5) Caregiver needs	66.74 (15.20)	23–100	0.03	−0.12	0.39**	0.36**	-		
(6) Family resilience	43.09 (5.50)	19–59	−0.07	−0.10	−0.36**	−0.44**	−0.28**	-	
(7) Disability numbers	3.19 (1.43)	1–8	0.05	0.05	0.18**	0.21**	0.23**	−0.10	-

**p < 0.01.

A stepwise multiple regression analysis was conducted to examine the relative contribution of various factors and their interactions. The results are summarized in Table 3. In Model 1, demographic variables such as the ages of parents and the target children, and the number of their disabilities were included. This model accounted for 3% of the variances in caregiver burnout, with the number of disabilities being the only significant factor (*b* = 0.40, *p* < 0.00, 95% CI [0.13, 0.68]). Model 2 included an individual factor of self-stigma, the family factor of family resilience, and the external factor of caregiver needs after controlling for demographic variables in Model 1. The inclusion of these variables in Model 2 explained an additional 26% of the variance in caregiver burnout. Self-stigma (*b* = 0.13, *p* < 0.01, 95% CI [0.08, 0.19]), family resilience (*b* = −0.09, *p* = 0.01, 95% CI [−0.16, −0.02]), and caregiver needs (*b* = 0.05, *p* < 0.01, 95% CI [0.02, 0.07]) were significant predictors, supporting Hypotheses 1 to 3. Model 3 was then conducted to examine the interplay among the three significant factors by including a 3-way interaction (self-stigma X family resilience X caregiver needs) and their respective 2-way interaction terms. This model accounted for an additional 2% of the variance in caregiver burden. Results showed a significant 3-way interaction effect (*b* = 0.01, *p* = 0.04, 95% CI [0.00, 0.001]), a significant 2-way interaction effect between self-stigma and caregiver needs (*b* = −0.02, *p* = 0.04, 95% CI [0.03, −0.001]), and a marginal significant interaction effect between family resilience and caregiver effect (*b* = −0.01, *p* = 0.05, 95% CI [−0.03, 0.001]). Overall, the final model explained 31% of the variance in caregiver burnout, demonstrating a complex interplay of various contributing factors and supporting Hypothesis 4.

**Table 3:** Multiple regression models for predicting caregiver burnout (*N* = 250).

Models	Caregiver burnout						
						95% CI	
	*b*	*b* SE	β	*t*	*p*	LL	UL
Model 1							
Age of participants	0.02	0.05	0.03	0.50	0.62	−0.07	0.11
Age of target children	−0.08	0.11	−0.05	−0.78	0.44	−0.29	0.13
Total number of disabilities	0.40	0.14	0.18	2.86	0.00	0.13	0.68
*R*^2^ = 0.034							
*F(3, 246) =* 2.92*							
Model 2							
Age of participants	−0.00	0.4	0.00	−0.00	1.00	−0.08	0.08
Age of target children	−0.03	0.09	−0.02	−0.34	0.74	−0.22	0.15
Total number of disabilities	0.12	0.13	0.05	0.93	0.36	−0.13	0.37
Self-stigma	0.13	0.03	0.30	4.80	0.00	0.08	0.19
Family resilience	−0.09	0.04	−0.16	−2.62	0.01	−0.16	−0.02
Caregiver needs	0.05	0.01	0.22	3.67	0.00	0.02	0.07
*R*^2^ = 0.29							
*F(6, 243)* = 16.74**							
Model 3							
Age of participants	−0.00	0.04	0.00	−0.03	0.98	−0.08	0.08
Age of target children	−0.03	0.09	−0.02	−0.29	0.77	−0.21	0.16
Total number of disabilities	0.10	0.13	0.05	0.81	0.42	−0.15	0.35
Self-stigma	1.38	0.71	3.12	1.93	0.05	−0.03	2.78
Family resilience	0.82	0.50	1.40	1.63	0.10	−0.17	1.81
Caregiver needs	0.60	0.29	2.85	2.10	0.04	0.04	1.16
Self-stigma x Family resilience	−0.03	0.02	−2.60	−1.77	0.08	−0.06	0.00
Self-stigma x Caregiver needs	−0.02	0.01	−4.18	−2.04	0.04	−0.03	0.001
Caregiver needs x Family resilience	−0.01	0.01	−2.70	−1.99	0.05	−0.03	0.00
Self-stigma x Family resilience x Caregiver needs	0.01	0.00	3.70	2.07	0.04	0.00	0.001
*R*^2^ = 0.31							
*F(10, 239)* = 10.61**							

*p < 0.05, **p < 0.01.

To further understand the significant interaction effects of the regression analyses, the median split method was adopted to derive high and low levels of the variables. The median split scores were 29 for self-stigma, 44 for family resilience, and 67 for caregiver needs. Participants scoring above the median were classified as having high levels of the variables, whereas those scoring at or below the median were classified as having low levels of the variables. It should be noted that the presence of a significant 3-way interaction indicates that the relationship between any two variables depends on the level of the third variable. Consequently, the effects of the 2-way interactions cannot be fully understood without considering the third variable, as these simpler 2-way interactions are not consistent across all levels of the third variable. Therefore, this study focused on the 3-way interaction effect that provided a more comprehensive understanding of the relationships among the variables. Figures 1A,B illustrates the interaction between family resilience and caregiver needs on caregiver burnout depended on the level of self-stigma. In the low-stigma condition, the highest level of caregiver burnout was found among mothers with low family resilience and high caregiver needs. On the other hand, in the high-stigma condition, the highest level of burnout was observed among mothers with high family resilience and high caregiver needs.

**Figure 1A:**
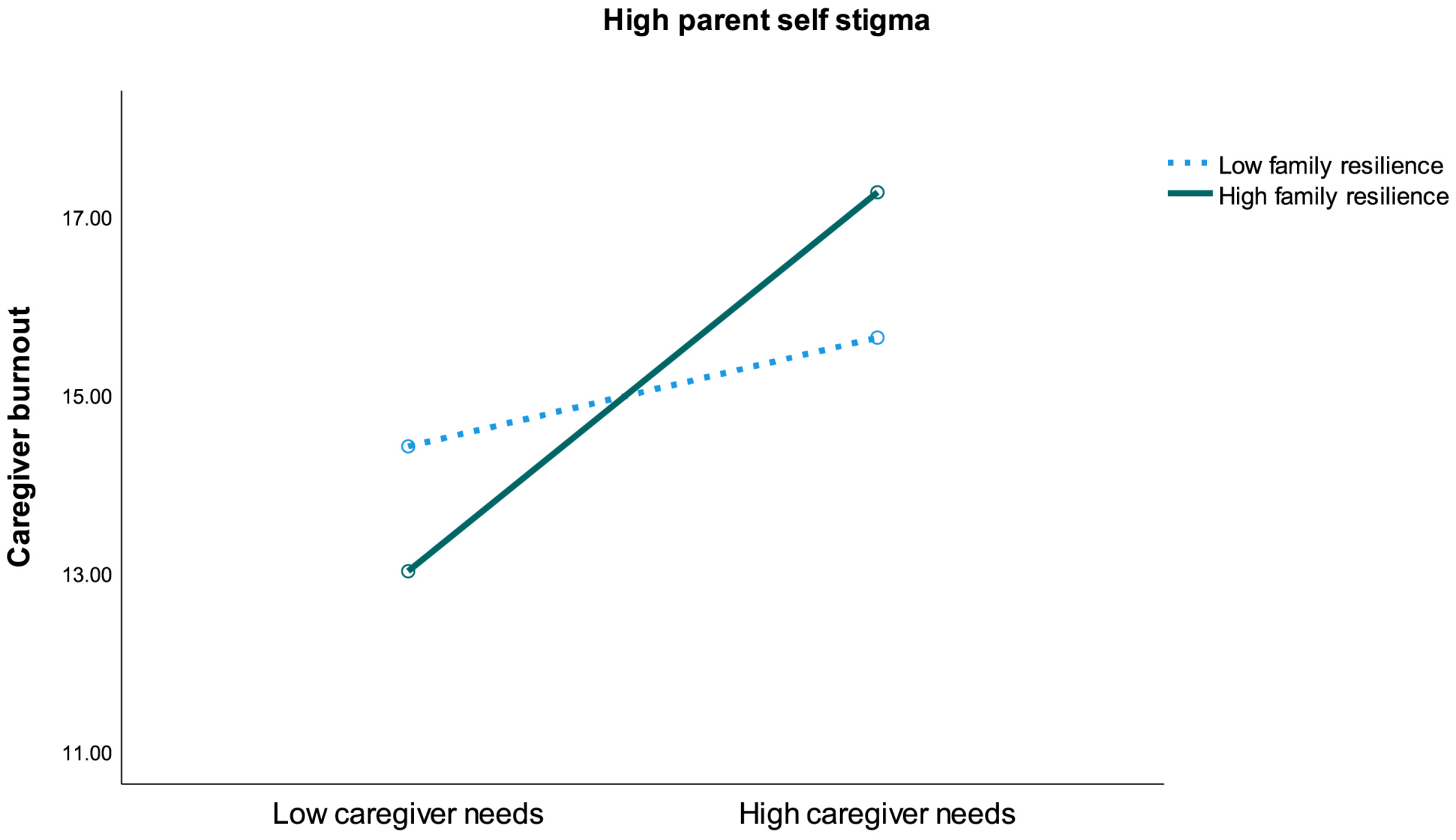
Interaction between family resilience and caregiver needs on caregiver burnout in high-stigma condition.

**Figure 1B:**
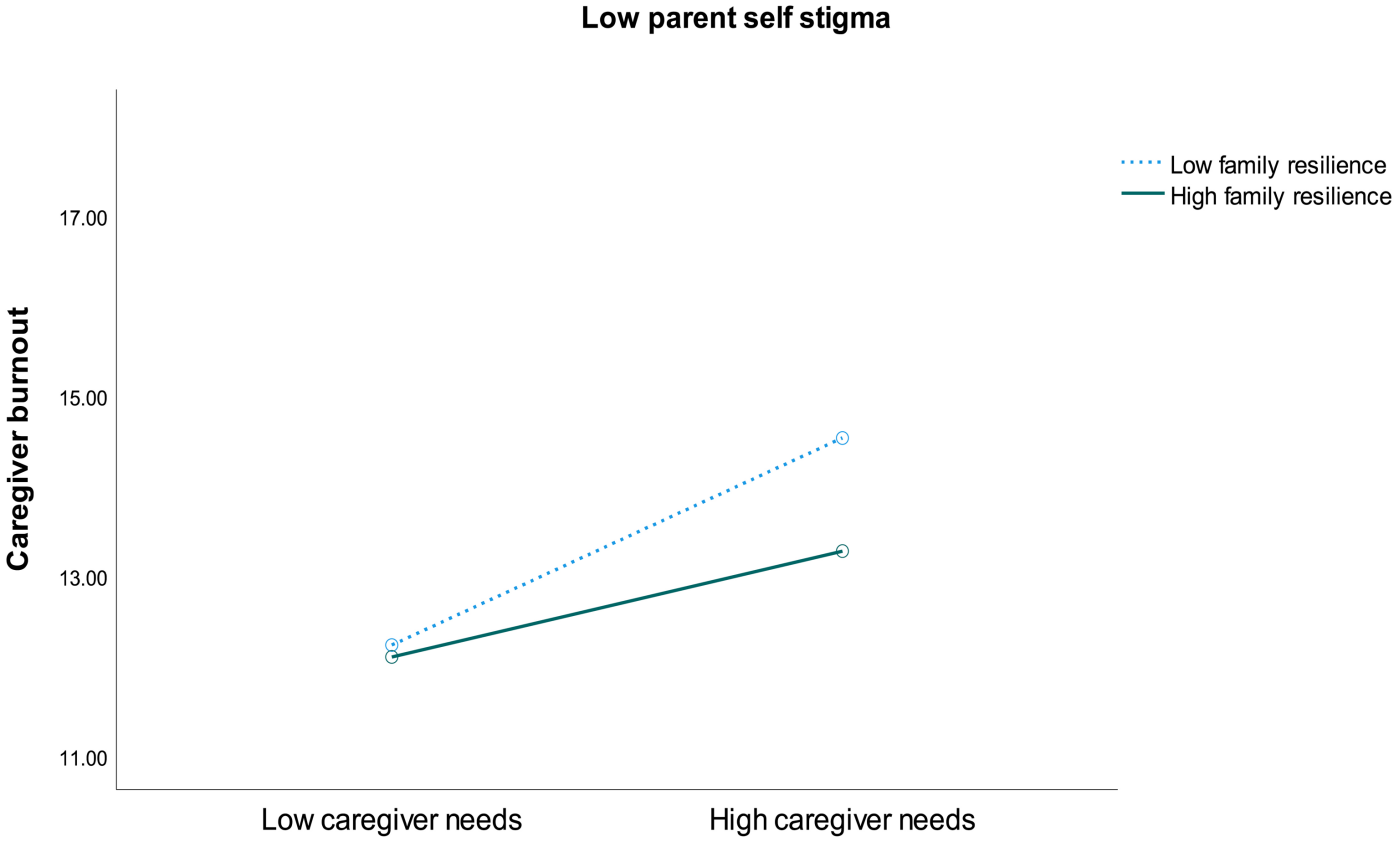
Interaction between family resilience and caregiver needs on caregiver burnout in low-stigma condition.

The regression analyses also revealed a significant 2-way interaction effect between self-stigma and caregiver needs on caregiver burnout (*b* = −0.02, *p* = 0.04, 95% CI [−0.03, −0.001]). This finding indicated that caregiver burnout increased with higher caregiver needs, with a more pronounced effect among caregivers with high self-stigma compared to those with low self-stigma. Additionally, a marginally significant 2-way interaction effect between family resilience and caregiver needs was also observed (*b* = −0.01, *p* = 0.05, 95% CI [−0.03, 0.001]). This result suggested that caregiver burnout increased with higher caregiver needs, with a more pronounced effect among caregivers with high family resilience compared to those with low family resilience.

## 4 Discussion

This study explored multifaceted factors and their interplay in understanding parental caregiving burnout in Hong Kong using an ecological framework (Bronfenbrenner, 1979; Bronfenbrenner and Evans, 2000). Findings supported the general hypotheses and indicated that high self-stigma, low family resilience, and unmet caregiver needs independently contribute to caregiving burnout of mothers with SN children. High levels of caregiver self-stigma is related to greater burnout, as caregivers internalize negative beliefs about themselves, feel shame and incompetence in their caregiving role, and experience emotional exhaustion (De Leersnyder et al., 2013; Eaton et al., 2019; Awan et al., 2022). This self-stigma exacerbates their stress and diminishes their capacity to cope, directly increasing the risk of burnout. Family resilience, on the other hand, generally acts as a protective factor. Higher levels of family resilience can buffer against the stress and challenges of caregiving by providing emotional support, effective communication, and problem-solving skills within the family unit (Cheng and Lai, 2023; Cici and Özdemir, 2024; Patty et al., 2024; Tang et al., 2024). Moreover, when caregivers’ needs are not met, they face additional stressors that compound their emotional and physical exhaustion (Neely-Barnes and Dia, 2008; Casagrande and Ingersoll, 2021; Rakap and Vural-Batik, 2023). High levels of unmet caregiver needs indicate that caregivers are not receiving the necessary support to manage their responsibilities effectively, making them vulnerable to caregiver burnout.

Consistent with previous studies, our findings highlight that caregiver burnout is related to a complex interplay of factors within the caregivers’ ecological system (Cheng and Lai, 2023; Abeasi et al., 2024; Lobo et al., 2024). Notably, a three-way interaction effect among the factors revealed that the highest levels of caregiver burnout were reported by mothers experiencing high self-stigma, high family resilience, and high unmet caregiving needs. Additionally, a two-way interaction effect indicated that the increase in caregiver burnout due to high unmet caregiving needs was more pronounced among mothers with high family resilience and mothers with high self-stigma. The intricate interplay of factors may be rooted in cultural and gender dynamics. In Chinese society, the concept of filial piety places significant emphasis on the duty to care for family members, a responsibility that often falls disproportionately on women (Shek et al., 2019; MacMullin et al., 2021; Zhang, 2022). This cultural expectation can amplify self-stigma among caregiving mothers of children with SN, as they may feel intense pressure to fulfil their roles and internalize any perceived shortcomings. While family resilience is generally protective, it can paradoxically increase pressure to conform to the cultural ideal of motherhood. High family resilience often implies strong support among family members, leading to expectations that mothers will manage their responsibilities without showing signs of stress or struggle. This pressure can be overwhelming, as mothers may feel compelled to meet their family’s high standards, making them reluctant to seek additional support in caregiving for their children with special needs (SN). This reluctance exacerbates stress and contributes to burnout. Furthermore, despite family support, mothers may perceive a lack of external resources from the community, increasing their stress and emotional exhaustion, especially when combined with high self-stigma. This situation is particularly relevant in Hong Kong, where mental health and support services for SN children are often inadequate and stigmatized, leaving many caregiver needs unmet. The intersection of cultural pressures, traditional female gender roles, and unmet needs creates a challenging environment that significantly heightens the risk of caregiver burnout among Chinese mothers of children with SN. In summary, while family resilience can offer protection, it can also create additional stress if mothers feel pressured to meet cultural ideals and are unable to access adequate external support. Addressing these issues requires a multifaceted approach that considers cultural, social, and systemic factors.

This study provides valuable insights; however, the clinical validity is somewhat constrained by various limitations. First, this study only included mother caregivers, as fathers are typically reluctant to participate in research and intervention programmes on child rearing and caregiving (Keys et al., 2019). Consequently, the experiences of fathers in Chinese families, who may face different challenges and stressors, are overlooked. Second, the age of the target children were limited to between 3 and 12 years, so the findings may not generalize to caregivers of children in other age groups. Third, participants who have access to and are comfortable with online surveys may differ in significant ways from those who do not, potentially limiting the generalizability of the findings. Fourth, the reliance on self-reported data can introduce social desirability and recall bias, as participants may underreport negative experiences or overreport positive behaviors, potentially influencing the associations among variables. Additionally, this cross-sectional study only provides relationship among variables at a single point in time and no conclusive cause-effect inferences can be made. Finally, this study does not examine the broader socio-economic and healthcare policy environment in Hong Kong, which can significantly impact caregiver burnout. Factors such as access to healthcare, the availability of public services, and economic pressures were not explored, despite their potential influence on caregiver stress.

To address the above limitations, future studies should include fathers by designing targeted outreach strategies such as flexible programme schedules and collaborations with father organizations. In the Chinese context, other extended family members such as grandparents can also be included to assess their support for caregiving. Expanding the age range of children can provide a comprehensive understanding of caregiver burnout across different developmental stages. In addition, future research should adopt a mixed-method approach combining both qualitative and quantitative designs. Diversifying data collection methods to include in-person interviews and phone surveys will reach participants with broader demographic characteristics and reduce biases related to internet access. Conducting longitudinal studies would allow for the assessment of causal relationships and changes over time. Lastly, examining the broader socio-economic and healthcare policy environment will help understand the impact of factors like access to healthcare services, public support programmes, and economic pressures on caregiver stress. By addressing these limitations, future research can offer a more detailed understanding of caregiver burnout to facilitate the design of more effective interventions.

Despite the aforementioned limitations, the findings of this study underscore the necessity for a holistic, culturally sensitive, and personalized approach in understanding and effectively reducing caregiving burnout among Chinese mothers of children with special needs (SN). The interaction effects among factors indicate that integrated support systems addressing multiple factors simultaneously are essential. High self-stigma significantly predicts caregiver burnout, especially when combined with high family resilience and high caregiver needs. Interventions should focus on reducing self-stigma among these mothers through cognitive-behavioral therapy (CBT), support groups, and psychoeducation. Family resilience is crucial in modulating the impact of unmet caregiver needs on burnout. Programmes aimed at strengthening family resilience, such as family therapy, resilience training, and community support initiatives, can help mitigate burnout by providing emotional and practical support to mother caregivers. It should be noted that while family resilience can be protective, it can also increase stress if high expectations are placed on mothers, leading them feeling the need to always appear strong and neglect their own needs. Thus, regularly assessing mothers’ needs and ensuring open communication with the family can help mitigate these challenges. Family members should set clear boundaries, prioritize communication, create a support schedule, and encourage mothers to seek professional guidance if necessary. High unmet caregiver needs are consistently associated with increased burnout, regardless of self-stigma and family resilience levels. As such, healthcare providers should identify and address these unmet needs, offering resources like respite care, financial assistance, and specialized healthcare services to reduce the burden on mother caregivers. Advocacy for policy changes that improve access to caregiver support services, healthcare, and financial aid is also crucial.

In conclusion, addressing caregiver burnout among Chinese mothers of children with SN requires a comprehensive and multifaceted approach. A more supportive and effective caregiving environment can be created by reducing self-stigma, enhancing family resilience, addressing unmet needs, and advocating for supportive policies.

## Data Availability

The raw data supporting the conclusions of this article will be made available by the authors, without undue reservation.
